# Analysis of the Clinical Education Situation framework: a tool for identifying the root cause of student failure in the United States

**DOI:** 10.3352/jeehp.2019.16.11

**Published:** 2019-05-10

**Authors:** Katherine Myers, Kyle Covington

**Affiliations:** Doctor of Physical Therapy Division, Duke University School of Medicine, Durham, NC, USA; Hallym University, Korea

**Keywords:** Educational measurement, Physical therapy specialty, Root cause analyses, United States

## Abstract

Doctor of physical therapy preparation requires extensive time in precepted clinical education, which involves multiple stakeholders. Student outcomes in clinical education are impacted by many factors, and, in the case of failure, it can be challenging to determine which factors played a primary role in the poor result. Using existing root-cause analysis processes, the authors developed and implemented a framework designed to identify the causes of student failure in clinical education. This framework, when applied to a specific student failure event, can be used to identify the factors that contributed to the situation and to reveal opportunities for improvement in both the clinical and academic environments. A root-cause analysis framework can help to drive change at the programmatic level, and future studies should focus on the framework’s application to a variety of clinical and didactic settings.

## Introduction

Approximately one-third of the curriculum of a doctor of physical therapy (DPT) program is composed of clinical education experiences [1]. The Commission on Accreditation in Physical Therapy Education requires DPT education programs to have a comprehensive plan to determine program effectiveness [2].

There are many stakeholders involved in clinical education, including the academic program and academic faculty, the student, the patient, and the clinical faculty [3]. The reasons for a student’s failure can also be complex and multifaceted. Furthermore, failure in a clinical education course can have a significant impact on the student’s progression in the DPT program and overall programmatic outcomes.

Root cause analysis (RCA) is one way in which programs may examine student failure in clinical education. RCA is utilized frequently in various fields, including healthcare and business, to examine a challenging or unsuccessful situation from multiple perspectives. The RCA process serves to understand what happened in a situation, why it happened, and what can be done to prevent similar situations from occurring again [4]. The analysis typically provides a big-picture perspective of the situation, as well as the associated details, and can facilitate corrective action. Ultimately, the intent of RCA is to go beyond identification of one ‘cause’ to reveal a ‘system of causes’ that impacted a situation [5].

Academic faculty at a DPT program drew from the RCA approach and developed the Analysis of Clinical Education Situations (ACES) framework as a mechanism for formal, objective analysis of student failure in DPT clinical education experiences. This framework examines student failure events from multiple perspectives to determine potential strategies to prevent similar situations from occurring again.

## Academic program description

Duke University’s DPT program includes a 3-year curriculum with 43 weeks of clinical education coursework. The third year of the program includes three 12-week full-time terminal clinical experiences. The full-time clinical experiences occur at clinical sites external to the academic program, and practicing clinicians employed by the clinical site serve as clinical instructors (CIs).

In each full-time clinical experience, CIs use the Clinical Performance Instrument (CPI) to evaluate student performance. The CPI is a validated assessment tool that includes 18 separate performance criteria with defined benchmarks. Student failure in clinical education is defined as the student not achieving the expected performance outcome of the clinical education course, which can include failure to meet benchmarks on the CPI, issues with professional behavior, or a combination of both. If deemed appropriate by the program, student failure in a clinical education course may result in the student repeating the clinical experience following an individualized remediation program. The program had an established process to determine whether a student could and should repeat a clinical experience, but there was no systematic process to review causes of student failure.

## Framework development and description

**Fig. 1.**
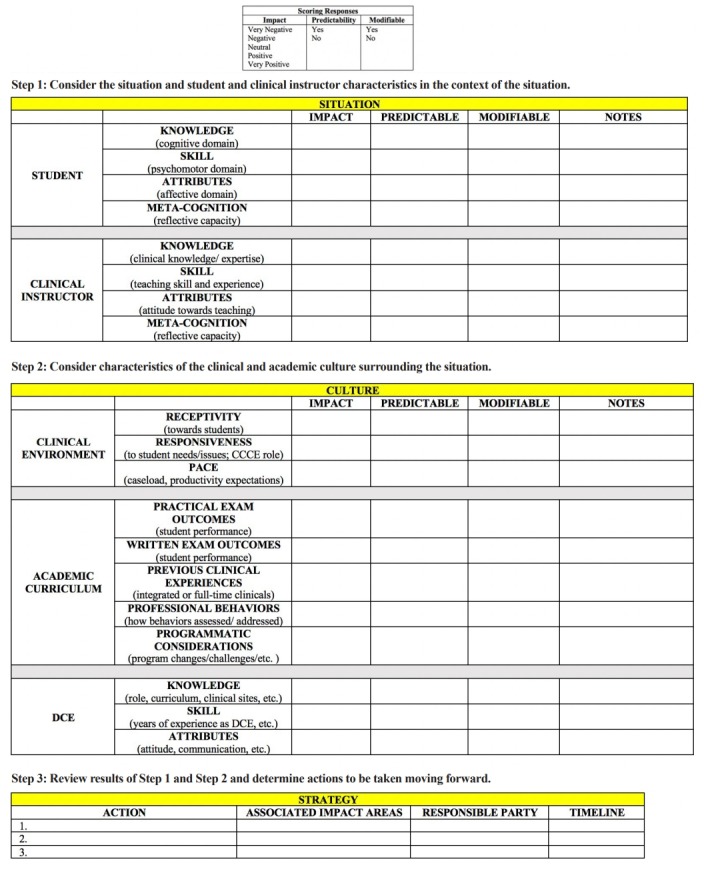
Analysis of Clinical Education Situations framework.

While the term ‘root cause analysis’ implies that there is one primary cause of an adverse event, the framework designers recognized that any process that analyzes challenges in clinical education must account for the multitude of factors that may impact such situations. Therefore, the authors aimed to create a process that would account for the complexities of clinical education. The ACES framework (Fig. 1) was developed based on existing RCA methods in healthcare and medical education [6-8]. The framework also considers factors specific to physical therapy education that have been shown to have a direct impact on the clinical learning experience. These include barriers and supports to learning related to the clinical environment and CI, as well as specific characteristics of the academic program and academic curriculum [9,10].

The ACES framework is a 3-step process that begins with an examination of the central situation of student failure, focusing on factors related to the student and CI. The process then broadens in step 2 to examine how the clinical environment, the academic curriculum, the academic program, and the director of clinical education (DCE) may have influenced the situation. Each factor in step 1 and step 2 is evaluated along 3 dimensions: impact, predictability, and modifiability. The first dimension, impact, utilizes a 5-point scale from very positive to very negative. Predictability and modifiability are dichotomous, with the response to each being “yes” or “no.” A characteristic is determined to be predictable if the academic program could have predicted the impact of this characteristic on the situation, and it is determined to be modifiable if the academic program could have modified or acted upon this characteristic at any point to change it in some way.

In the final step, the framework user is prompted to review each characteristic. Analysis of the characteristics that have the greatest impact and are predictable and/or modifiable should result in actions or strategies to address these root cause(s). Accountability is built into the framework by the specific identification of responsible parties and a timeline for completion of the action.

## Sample application of the Analysis of Clinical Education Situations framework

### Case description

A DPT student completed all on-campus didactic and integrated clinical education experiences during the first 2 years of the program. In the third year of the program, the student successfully completed the first 12-week full-time clinical experience in an outpatient orthopedic practice by achieving the prescribed advanced intermediate level on the CPI evaluation. The student began a second 12-week full-time clinical experience at an inpatient rehabilitation facility.

During the fifth week, the student and the CI reached out to the academic program’s DCE to discuss performance concerns. From that point forward, the DCE was in frequent contact via email and phone with the student, the CI, and the Site Coordinator of Clinical Education (SCCE). The DCE spoke at least twice a week with the student, and the phone calls included coaching on clinical reasoning, communication strategies, and self-reflection. The DCE received weekly updates from the CI by email and phone regarding the student’s performance in the clinic that week, areas of improvement, and areas of continued challenge. The DCE performed a site visit in week 8 that included observation of the student and CI during a patient encounter and during a post-encounter debriefing session. Following the site visit, the DCE, CI, SCCE, and student established a learning plan and specific learning objectives. Ultimately, the clinical experience was terminated in week 10 due to significant and ongoing concerns regarding safety and clinical reasoning. The student received a failing grade for the clinical education course.

### Framework application

**Fig. 2.**
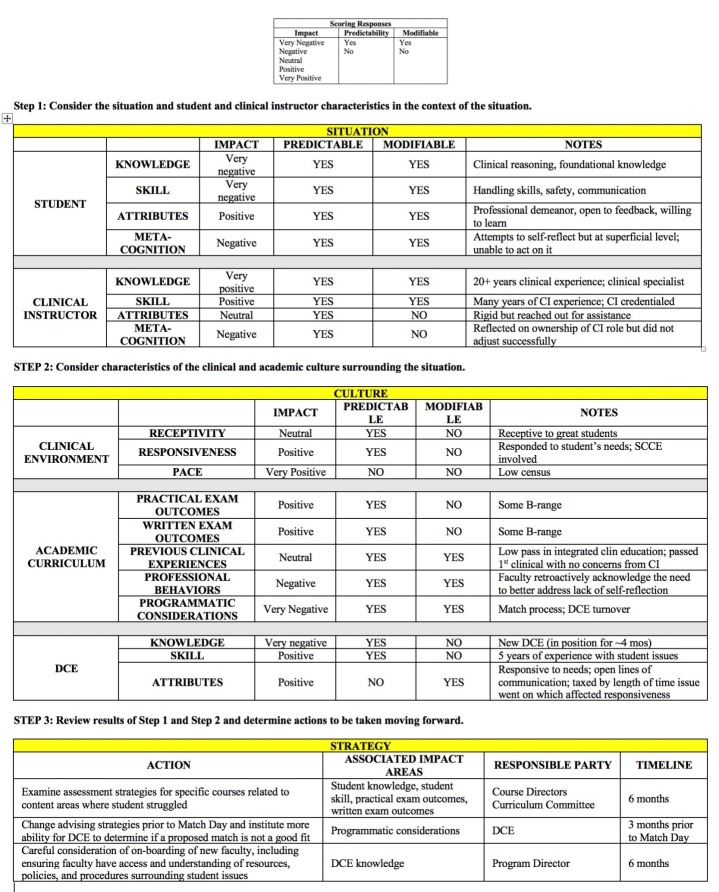
Student example: analysis of Clinical Education Situations framework.

The ACES framework was applied to this student’s case (Fig. 2). The analysis of impact, predictability, and modifiability within step 1 and step 2 revealed that student characteristics and academic curriculum considerations were the main contributing factors. There was a disconnect between the student’s knowledge and skills (negative impact) and the outcomes of the written and practical exams (positive impact). Further analysis of the framework revealed an opportunity for improvement around programmatic processes, including assessment of clinical reasoning, how students are matched to clinical sites, and how new faculty are onboarded and introduced to policies, procedures, and resources.

The action plan detailed in step 3 addressed the curriculum factors and programmatic processes. The curriculum committee and course directors reviewed the current assessment strategies in core coursework related to clinical reasoning. The DCE reviewed advising strategies and clinical site matching processes to address the concern that, in this case, the student and clinical site were not a good fit. Finally, the program leadership recognized a need for more specific faculty onboarding procedures, as well as gaps within the current policies and procedures related to management of student progression issues.

### Merits of the analysis of Clinical Education Situations framework

The use of the ACES framework within the authors’ academic program has resulted in a formalized mechanism to evaluate student failure situations and to describe programmatic outcomes at a more detailed level. The approach provides a needed mechanism to evaluate the clinical education program through a multifaceted approach that accounts for all stakeholders. The sample case is an example of how this process can result in avenues for greater academic-clinical partnership, as the RCA process may lead to collaborative efforts between academic and clinical stakeholders to address opportunities for program refinement.

The important impact of the ACES framework results from the action recommendations determined in step 3. The United States Department of Veterans Affairs National Center for Safety developed an ‘Action Hierarchy’ for RCA processes in 2001. This hierarchy categorizes action recommendations based on their effectiveness and sustainability, using the classifications of strong, intermediate, and weak [11]. The Institute for Healthcare Improvement advocates that recommendations from any RCA process should include at least one strong or intermediate-strength action [4]. When applying this hierarchy to the case described in this paper, the actions were found to fall in the intermediate and weak categories. Integration of this type of classification system into the final step of the ACES framework may result in more sustainable and impactful actions.

Further use of the framework and formalized study of its applicability are necessary to determine its effectiveness, reliability, and validity in consistently identifying the root problems in clinical education situations. This would include application of the framework within a variety of academic programs and clinical situations, potentially allowing for further refinement of the tool to maximize its effectiveness. The ACES framework also has the potential to be applied to other student situations beyond clinical education, resulting in a formalized and structured programmatic evaluation mechanism. However, applications in the didactic setting may require an altered version of the framework, as there are different influences, factors, and characteristics to consider in the classroom setting. Ultimately, the authors of the framework intend to utilize the framework in a multi-institutional study that aims to examine trends in clinical education situations across institutions.

## Conclusion

The use of a structured and multifaceted approach to analyzing clinical education problems may provide programs with a standardized way to assess situations and make changes to prevent repeat issues. Furthermore, the use of ACES may also provide programs with a tool for organizing discussion and collaboration among the stakeholders involved in clinical education. By implementing standardized approaches to program evaluation that result in opportunities for change, programs can reduce the time and effort involved in ongoing problem mitigation and student remediation.

## Supplementary materials

Supplement 1. Audio recording of the abstract.
